# Dengue virus reduces expression of low-density lipoprotein receptor-related protein 1 to facilitate replication in *Aedes aegypti*

**DOI:** 10.1038/s41598-019-42803-9

**Published:** 2019-04-23

**Authors:** Maya O. Tree, Berlin Londono-Renteria, Andrea Troupin, Kellie M. Clark, Tonya M. Colpitts, Michael J. Conway

**Affiliations:** 1 Foundational Sciences, Central Michigan University, College of Medicine, Mount Pleasant, MI United States of America; 2 Department of Pathology, Microbiology and Immunology, University of South Carolina School of Medicine, Columbia, South Carolina United States of America; 3 Department of Entomology, Kansas State University, Manhattan, Kansas United States of America; 4 Department of Microbiology, National Emerging Infectious Diseases Laboratories, Boston University School of Medicine, Boston, MA United States of America

**Keywords:** Dengue virus, Viral vectors

## Abstract

*Aedes aegypti* is the primary vector of a number of viruses pathogenic to humans including dengue virus (DENV). DENV infection leads to widespread transcriptomic and proteomic alterations in mosquito cells. Here we identified alterations to the mosquito cell secretome during DENV infection by performing liquid chromatography tandem mass spectrometry. We found that an extracellular fragment of low-density lipoprotein receptor-related protein 1 (LRP-1) was present during infection. Previous literature suggests that LRP-1 regulates cholesterol homeostasis. Therefore, we hypothesized that DENV modifies LRP-1 protein expression to maintain host-derived intracellular cholesterol, which would facilitate virus replication within membrane-associated replication compartments. Accordingly, stimuli that are present during flavivirus infection reduced LRP-1 protein expression. We also found that dsRNA knockdown of LRP-1 increased intracellular cholesterol and DENV viral RNA. Further, depletion of intracellular lipids reduced infection. Together, these data suggest that DENV reduces LRP-1 protein expression, possibly through regulated intramembrane proteolysis (RIP), to increase intracellular cholesterol and facilitate replication in *Ae. aegypti*.

## Introduction

*Aedes aegypti* is the primary vector of a growing list of pathogenic viruses that cause disease in humans including dengue virus (DENV). DENV infection leads to multiple clinical manifestations ranging from a febrile illness called dengue fever to severe dengue, which can include septic shock^1,2^. There are no targeted antiviral therapies for DENV and use of a conventional vaccine may risk causing antibody-dependent enhancement of DENV or a related pathogenic flavivirus^3^. It is theoretically possible to target vector proteins or cellular pathways as a method to interfere with the flavivirus transmission cycle^3–11^. This strategy requires identification of proteins or cellular pathways that modify flavivirus acquisition or transmission.

Flavivirus acquisition in the mosquito begins when the mosquito engorges on the blood of an infected vertebrate host. Virions establish infection of midgut epithelial cells and infection leads to widespread transcriptomic and proteomic alterations^4,12^. We hypothesized that flavivirus infection alters the mosquito cell secretome – the proteins and peptides that are secreted into the extracellular milieu and play important pathological and physiological roles. Characterizing the secretome can identify key proteins and cellular pathways that are modified during infection and reveal new therapeutic targets^13–15^.

Here we find that DENV infection of *Ae. aegypti* cells leads to accumulation of extracellular fragments of low-density lipoprotein receptor-related protein 1 (LRP-1). LRP-1 belongs to a group of cell surface proteins that can undergo regulated intramembrane proteolysis (RIP) upon ligand binding in a Notch-like fashion. LRP-1 proteolysis leads to the release of both intracellular and extracellular domains. The intracellular domain mediates signal transduction. The role of the soluble extracellular domain is less clear^16^.

Previous literature suggests that LRP-1 is a multifunctional cell surface receptor with diverse physiological roles ranging from cellular uptake of lipoproteins and other cargo to promoting cholesterol export and inhibiting accumulation of intracellular cholesterol^17–24^. LRP-1 deficiency has led to intracellular lipid accumulation in a number of experimental models^17–24^. In the context of cytomegalovirus (CMV) infection, LRP-1 inhibits virion infectivity by depleting intracellular cholesterol, which limits the amount of cholesterol in the viral envelope^25^. We hypothesized that DENV infection modifies LRP-1 protein expression, which increases host-derived intracellular cholesterol - a requirement for synthesis of intracellular membrane replication compartments.

## Materials and Methods

### Cell culture and virus production

Two *Aedes spp*. cell lines were used in this study, Aag2 and C6/36 cells. The *Ae. aegypti* cell line, Aag2 (ATCC, VA), was used for *in vitro* studies. Aag2 cells were grown at 28 °C with 5% CO2 in Schneider’s Drosophila Medium for transfection studies and DMEM high glucose media for the remaining studies. Both types of media were supplemented with 10% heat-inactivated fetal bovine serum (Gemini, CA), 1% penicillin-streptomycin, and 1% tryptose phosphate broth (Sigma, MO). Lipid-depleted serum was made by incubating serum with fumed silica overnight followed by removal of silica-lipid complexes by centrifugation^26^. Lipid-depleted serum was added to cell culture components to make lipid-depleted media. In addition, the *Ae. albopictus* cell line, C6/36 (obtained from Erol Fikrig), was used to grow DENV stocks using the same media. The dengue strain used was DENV-2 New Guinea C. C6/36 cells were infected at an MOI of 1.0 virus stock was stored at −80 °C until use.

### LC + MS/MS

Cell free supernatants were taken from mock and DENV2-infected Aag2 cells at 1 and 7 dpi and submitted to the Interdisciplinary Center for Biotechnology Research at the University of Florida for liquid chromatography tandem mass spectrometry (LC + MS/MS). Charge state deconvolution and deisotoping were not performed. All MS/MS samples were analyzed using Mascot. Mascot was set up to search the Aedesaegypti_201505 database (selected for *Aedes aegypti*, unknown version, 37,800 entries) assuming the digestion enzyme trypsin. Mascot was searched with a fragment ion mass tolerance of 0.050 Da and a parent ion tolerance of 10.0 PPM. Carbamidomethyl of cysteine was specified in Mascot as a fixed modification. Gln- > pyro-Glu of the n-terminus, deamidated of asparagine and glutamine and oxidation of methionine were specified in Mascot as variable modifications. Scaffold (version Scaffold_4.4.8, Proteome Software Inc., Portland, OR) was used to validate MS/MS based peptide and protein identifications. Peptide identifications were accepted if they could be established at greater than 50.0% probability by the Peptide Prophet algorithm with Scaffold delta-mass correction. Protein identifications were accepted if they could be established at greater than 50.0% probability and contained at least 1 identified peptide. Protein probabilities were assigned by the Protein Prophet algorithm.

### qRT-PCR-based assays

RNA from Aag2 cells was isolated using RNeasy kit (Qiagen, CA) following the manufacturer’s instructions and RNA was kept at −20 °C until testing. The qRT-PCR assay was done using the QuantiFast kit according to the manufacturer’s instructions (Qiagen, CA). Oligos for the qRT-PCR reactions were: DENV-2 envelope (E) protein: F: 5′-CATTCCAAGTGAGAATCTCTTTGTCA-3′ R: 5′-CAGATCTCTGATGAATAACCAACG-3′; *Ae. aegypti* Actin: F: 5′-GAACACCCAGTCCTGCTGACA-3′, R: 5′-TGCGTCATCTTCTCACGGTTAG-3′. *Ae. aegypti* LRP-1: 5′-3′ F: 5′-GAAACAACCGGAAACAGCTCACCCATGTCG-3′ R: 5′-GATCCCCAGTGGAATGCCTCGGATCATGCC-3′. Viral RNA or LRP-1 expression was normalized to either total cellular RNA or actin expression. Each sample was tested in duplicate.

### dsRNA production and transfection

For gene knockdown, dsRNA was produced from approximately 500 bp coding regions of either *Ae. aegypti* LRP-1 (VectorBase: AAEL007041, GenBank: EAT41289) or green fluorescent protein (GFP) as a control. Briefly, PCR was used to produce a DNA template with T7 overhangs that was then used with the Ambion Megascript kit according to manufacturer’s instructions to produce the dsRNA molecules. Oligos used for making dsRNA are as follows: GFP: F: 5′-TAATACGACTCACTATAGGGAGAGATGCCACCTACGGCAAGC-3′ R: 5′-TAATACGACTCACTATAGGGAGAGACAGGTAGTGGTTATCGGG-3′LRP-1: F: 5′-TAATACGACTCACTATAGGGAGAatcgacgtttcggacgaagacgcggacc-3′R: 5′-TAATACGACTCACTATAGGGAGATTAATGCTAGGTGAGTGTTCGTgttggttac-3′. The dsRNA molecules were transfected into cells using Lipofectamine 2000 (ThermoFisher) according to manufacturer’s instructions.

### Intracellular cholesterol content

Total intracellular cholesterol was measured using the Amplex Red Cholesterol Assay Kit (Invitrogen). Aag2 cells were lysed in PBS containing 1% Triton X-100 and protease inhibitor cocktail. 2 ug of total protein was used for each sample. The reactions were brought up to a total reaction volume of 50 ul using 1x reaction buffer from the kit and applied to individual wells on a 96-well microplate. 50 ul of Amplex Red reagent/HRP/cholesterol oxidase/cholesterol esterase working solution was added to each well and the plate was incubated at 37 °C for 30 minutes protected from light. The fluorescence was measured using a Tecan Infinite M200 Pro microplate reader using excitation at 550 nm and emission detection at 590 nm.

### Western blot

Aag2 cells were lysed using RIPA buffer containing a protease inhibitor cocktail (Sigma). Protein concentration was determined using a BCA assay. 9 µg of total protein from each sample was separated by a SDS-PAGE gel (5–9%) and transferred to a nitrocellulose membrane. Membranes were blocked with 5% non-fat dry milk at room temperature for 1 hour prior to overnight incubation at 4 °C with primary antibody anti-LRP1 (cat. no. ab 92544, 1:20000, Abcam) and anti-actin antibody (cat. No. SC-1615, 1:1000, Santa Cruz Biotechnology) as a loading control. The membranes were washed with PBST and incubated at room temperature for 1 hour with anti-rabbit IgG-HRP (cat. no. 70745, 1:2000, Cell Signaling Technology) and anti-goat IgG-HRP linked (cat. no. ab6741, 1:2000, Abcam) secondary antibodies. Signal was detected using a ChemiDoc Touch Imaging System by Biorad.

### Focus-forming unit assay

Aag2 cells were fed with complete or lipid-depleted media for 7 days and then inoculated with 50 focus-forming units of DENV2 in complete media. Unbound virus was removed after one hour and complete media was added. Confluent monolayers were fixed with 4% paraformaldehyde and stained with 1:200 anti-DENV2 envelope antibody (3H5-1, EMD Millipore) 3 days post-infection and the total number of flavivirus positive foci were counted per well. Flavivirus positive foci were revealed using a 1:2000 horseradish peroxidase-conjugated secondary antibody and a Vectastain ABC kit immunocytochemistry kit. Digital images were taken using an Evos Core inverted microscope.

## Results

### Alterations of the Aag2 secretome during DENV infection

**Figure 1:**
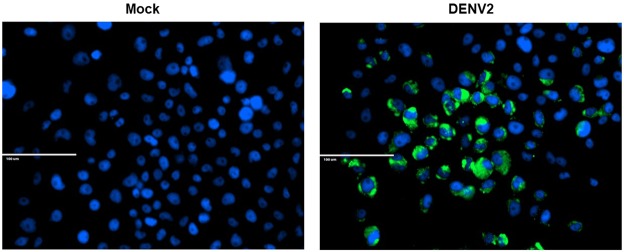
Immunocytochemistry of DENV2-infected Aag2 cells. Aag2 cells were grown in glass chambers and were inoculated with virus free (Mock) or DENV2-infected cell culture media and then fixed 7 dpi. Fixed cells were incubated with anti-DENV2 E antibody followed by a secondary antibody containing a fluorophore (green). Cell nuclei were counterstained with DAPI. Images were taken at 100x magnification.

**Figure 2:**
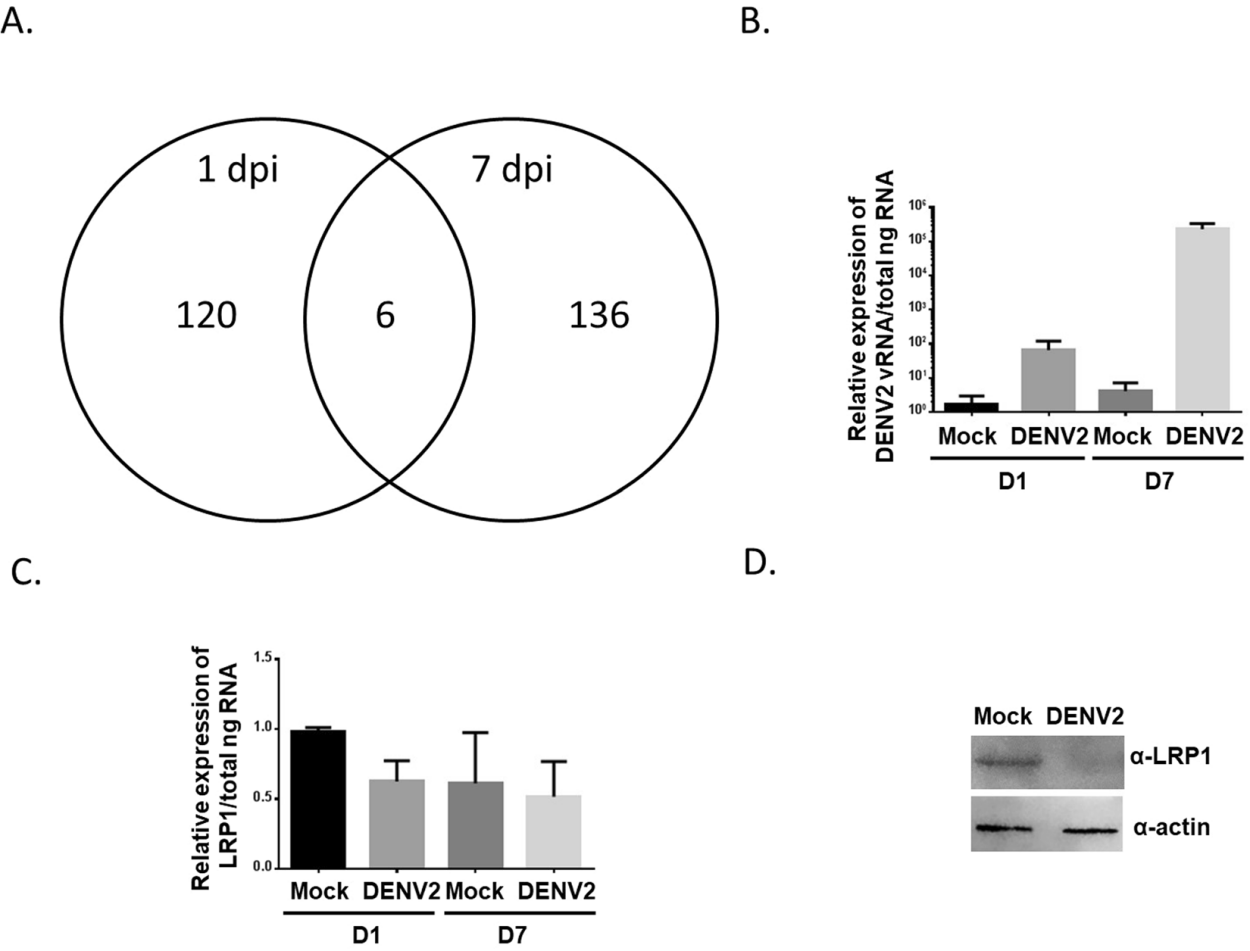
DENV2 infection induces shedding of LRP-1 peptides in Aag2 cells. (**A**) Venn diagram showing DENV2-specific proteins in the Aag2 secretome that were identified by LC + MS/MS 1 and 7 dpi. (**B**) qRT-PCR analysis of DENV2 viral RNA (vRNA) in cell culture supernatant in Mock and DENV2-infected cells 1 and 7 dpi. vRNA levels were normalized to total ng cellular RNA extracted from the cells in each well. (**C**) qRT-PCR analysis of LRP-1 mRNA in Mock and DENV2-infected cells 1 and 7 dpi. vRNA levels were normalized to total ng cellular RNA extracted from the cells in each well. (**D**) Western blot analysis of LRP-1 and actin in Aag2 cell lysates made from Mock and DENV2-infected cells 7 dpi.

We hypothesized that DENV infection would alter the secretome of mosquito cells. To test this hypothesis, we first confirmed previous results that the *Ae. aegypti* Aag2 cell line was permissive to DENV2 infection by inoculating cells with or without 1 × 10^6^ genome equivalents of DENV2 New Guinea Strain C. Cells were fixed 7 days post infection (dpi) and stained with an anti-DENV2 envelope monoclonal antibody and secondary antibody conjugated with Alexa Fluor 488. Aag2 cells were highly permissible to infection and DENV2 envelope protein was observed in almost every cell (Fig. 1). Aag2 cells were then inoculated with approximately 1 × 10^6^ genomic equivalents of DENV2 for 1 hr, unbound virus was removed, and fresh media was added. Mock-infected controls were included. Cell-free supernatants were collected 1 and 7 dpi and submitted for liquid chromatography tandem mass spectrometry (LC + MS/MS) to detect the proteins in the secretome. We identified a total of 239 and 120 proteins in mock and DENV2-infected supernatants at 1 dpi, respectively. We identified a total of 183 and 136 proteins in mock and DENV2-infected supernatants at 7 dpi, respectively. The majority of proteins detected during infection were unique compared to mock infected samples. Approximately 80% of proteins detected in DENV-infected Aag2 cell supernatants were not identified in mock-infected supernatants at 1 or 7 dpi (Tables 1 and 2, Fig. 2A). Only 6 proteins were detected in DENV2-infected Aag2 cell supernatants but not mock-infected supernatants at both 1 and 7 dpi (Table 3, Fig. 2A).

**Table 1:** LC + MS/MS hits specific to DENV2-infected AaG2 cell supernatant 1 dpi.

Identifier	Comment
AAEL009437	N/A
AAEL012951	transformation/transcription domain-associated protein
AAEL007132	dynein heavy chain
AAEL007041	low density lipoprotein receptor (ldl)
AAEL012437	N/A
AAEL000981	N/A
AAEL007158	nnp-1 protein (novel nuclear protein 1) (nop52)
AAEL010871	N/A
AAEL000430	N/A
AAEL000895	peroxisome biogenesis factor 1 (peroxin-1)
AAEL004699	N/A
AAEL009431	mitochondrial ribosomal protein, L10, putative
AAEL011239	short-chain dehydrogenase
AAEL005240	rabaptin-5, putative
AAEL013174	N/A
AAEL013060	N/A
AAEL008220	N/A
AAEL017221	odorant receptor
gi|167515086	sodium-dependent nutrient amino acid transporter 8
AAEL007980	N/A
AAEL011526	GPCR Methuselah Family
AAEL003575	N/A
AAEL000589	serine/threonine protein kinase
AAEL001607	galactose-1-phosphate uridylyltransferase
AAEL011693	mitotic control protein dis3
AAEL010459	N/A
AAEL011810	N/A
AAEL009382	lysine-specific demethylase NO66
AAEL006340	N/A
AAEL000645	N/A
AAEL009477	N/A
AAEL015352	lachesin, putative
AAEL006765	N/A
AAEL003020	tailless (tll)
AAEL012825	bifunctional purine biosynthesis protein
AAEL005319	myosin light chain kinase
AAEL001796	nuclear hormone receptor (HR78)
AAEL006708	hedgehog
AAEL010693	N/A
AAEL004064	meiotic checkpoint regulator cut4
AAEL002508	26S protease regulatory subunit 6a
AAEL001716	THO complex subunit 2 (Tho2)
AAEL002544	N/A
AAEL006352	N/A
AAEL002628	N/A
AAEL004600	N/A
AAEL003168	N/A
AAEL011965	nuclear lamin L1 alpha, putative
AAEL014144	rapsynoid
AAEL006247	N/A
AAEL001442	map-kinase activating death domain protein
AAEL012744	N/A
AAEL013473	cAMP-specific 3,5-cyclic phosphodiesterase
AAEL000596	myosin
AAEL004376	N/A
AAEL013466	ankyrin 2,3/unc44
AAEL009159	nervous wreck, putative
AAEL009847	microtubule-associated protein
AAEL001582	kinesin
AAEL007079	N/A
AAEL006958	cell adhesion molecule
AAEL004299	angiotensin-converting-relatedenzyme
AAEL002896	N/A
AAEL014296	major sperm protein
AAEL008559	glutaminase
AAEL012158	WD repeat-containing protein 48
AAEL003008	N/A
AAEL007857	N/A
AAEL008610	myosin vii
AAEL005681	GPCR Histamine Family
AAEL006187	translational activator gcn1
AAEL009878	N/A
AAEL009019	N/A
AAEL012072	histone H3
AAEL009762	cytochrome P450
AAEL003641	sodium/chloride dependent amino acid transporter
AAEL001598	N/A
AAEL006806	N/A
AAEL011187	U520
AAEL015365	N/A
AAEL008591	zinc finger protein, putative
AAEL004644	N/A
AAEL008704	N/A
AAEL010473	NAD dependent epimerase/dehydratase
AAEL008365	N/A
AAEL014885	N/A
gi|70907192	N/A
AAEL013903	gamma-tubulin complex component 2 (gcp-2)
AAEL009480	N/A
AAEL009152	N/A
AAEL006050	nuclear RNA export factor 2 (NXF2), putative
AAEL003229	N/A
AAEL009049	N/A
AAEL000489	P21-activated kinase, pak
AAEL014315	N/A
AAEL010575	dimethyladenosine transferase
AAEL004049	N/A
AAEL017063	gustatory receptor Gr32
AAEL007565	leucine rich protein, putative
AAEL006394	N/A
AAEL002814	N/A
AAEL015467	segmentation protein cap’n’collar
AAEL002256	collagen alpha 1(xviii) chain
AAEL012774	protease m1 zinc metalloprotease
AAEL002850	patched 1
AAEL000318	N/A
AAEL000168	N/A
AAEL011566	cell adhesion molecule
AAEL001773	N/A
AAEL004320	WOC protein, putative
AAEL007400	signal recognition particle 54 kda protein
AAEL006067	N/A
AAEL012483	N/A
AAEL014664	AMP dependent coa ligase
AAEL003829	dynamin
AAEL001113	inorganic phosphate cotransporter, putative
AAEL013085	N/A
AAEL011231	N/A
AAEL010765	N/A
AAEL010308	N/A

**Table 2:** LC + MS/MS hits specific to DENV2-infected AaG2 cell supernatant 7 dpi.

Identifier	Comment
AAEL008845	N/A
AAEL001454	N/A
AAEL004179	N/A
AAEL007225	dynein heavy chain
AAEL011083	N/A
AAEL007132	dynein heavy chain
AAEL013943	mediator complex, 100kD-subunit, putative
AAEL015065	spectrin
AAEL013690	DNA mismatch repair protein pms2
AAEL006309	N/A
AAEL006374	N/A
AAEL006954	N/A
AAEL008154	N/A
AAEL000510	N/A
AAEL011103	centromere/kinetochore protein zw10
AAEL010342	racGTPase-activating protein
AAEL007099	environmental stress-induced protein, putative
AAEL011433	small nuclear ribonucleoprotein sm d3
AAEL009220	N/A
AAEL001581	N/A
AAEL000608	N/A
AAEL008318	cadherin, putative
gi|167651374	phosphoglycerate kinase, partial
AAEL011178	posterior sex combs protein
AAEL005881	autocrine motility factor receptor, amfr
AAEL002101	N/A
AAEL012446	Inhibitor of Apoptosis (IAP) containing Baculoviral IAP Repeat(s)
AAEL012338	N/A
AAEL003044	fasciclin-1 precursor
AAEL013593	N/A
AAEL009660	N/A
AAEL009839	nuclear cap-binding protein subunit 1
AAEL001921	N/A
AAEL001201	N/A
AAEL009308	WD-repeat protein
AAEL012587	N/A
AAEL013227	PIWI
AAEL002782	N/A
AAEL001357	N/A
AAEL006790	N/A
AAEL002250	terminal deoxycytidyl transferase rev1
AAEL004834	PHD finger protein
AAEL008670	protein tara
AAEL006082	transcription initiation factor TFIID subunit 1
AAEL010336	zinc phosphodiesterase
AAEL005211	microtubule associated-protein orbit
AAEL000668	N/A
AAEL002551	DNA topoisomerase type I
AAEL005284	receptor tyrosine phosphatase type r2a
AAEL003739	M-type 9 protein, putative
AAEL008573	zinc finger protein
AAEL006339	peptidylglycine alpha-amidating monooxygenase C-terminal protein-1
AAEL002670	AMP dependent ligase
AAEL006808	N/A
AAEL002070	N/A
AAEL013110	N/A
AAEL005381	dissatisfaction (Dsf)
AAEL001438	N/A
AAEL011052	N/A
AAEL004017	DNA polymerase v
AAEL015631	asparagine synthetase
AAEL014138	serine protease inhibitor (serpin)
AAEL005450	N/A
AAEL003105	supervillin
AAEL000007	N/A
AAEL000596	myosin
AAEL010061	N/A
AAEL007111	N/A
AAEL001411	myosin heavy chain, nonmuscle or smooth muscle
AAEL012331	N/A
AAEL005484	sorting nexin
AAEL013534	tgf-beta resistance-associated protein trag
AAEL005180	N/A
AAEL014995	ornithine decarboxylase
AAEL013559	uncoordinated protein
AAEL013561	N/A
AAEL004839	cyclin t
AAEL013635	N/A
AAEL005949	beta-1,2-n-acetylglucosaminyltransferase ii
AAEL004136	N/A
AAEL009164	tRNA delta(2)-isopentenylpyrophosphate transferase
AAEL004219	rap GTPase-activating protein
AAEL010940	N/A
AAEL010916	N/A
AAEL009673	GPCR Gonadotrophin Releasing Hormone Family
AAEL010101	N/A
AAEL007041	low density lipoprotein receptor (ldl)
AAEL013999	N/A
AAEL007447	N/A
gi|90655947	female specific 1 protein
AAEL011551	N/A
AAEL009958	N/A
AAEL005101	carboxy/choline esterase alpha esterase
AAEL008943	chromatin assembly factor 1, p180-subunit, putative
AAEL012223	zinc finger protein
AAEL008462	apl5 protein
AAEL003939	eukaryotic translation initiation factor 2-alpha kinase 3
AAEL010341	RuvB-like helicase 2
AAEL000912	N/A
AAEL004854	N/A
AAEL001375	Y-box binding protein
AAEL005461	N/A
AAEL002357	N/A
AAEL008358	ablim
AAEL002833	cathepsin l
AAEL012412	slit protein
AAEL007820	N/A
AAEL000423	N/A
AAEL011791	N/A
AAEL008653	engulfment and cell motility protein
AAEL004917	N/A
AAEL001649	leucine aminopeptidase
AAEL002922	glutamate receptor 7
AAEL008929	C-Type Lectin (CTL)
AAEL002216	5-AMP-activated protein kinase, beta subunit
gi|94468626	C2H2-type Zn-finger protein
AAEL007042	far upstream (fuse) binding protein
AAEL003345	argininosuccinate lyase
AAEL015537	N/A
AAEL001773	N/A
AAEL004196	galectin
AAEL009415	synembryn
AAEL013404	tetraspanin
AAEL011570	acidic fibroblast growth factor intracellular binding protein
AAEL000854	fumarylacetoacetate hydrolase
AAEL013729	myotonin-protein kinase
AAEL004880	N/A
AAEL004471	N/A
AAEL013174	N/A
AAEL002544	N/A
AAEL008075	N/A
AAEL002485	WD repeat-containing protein on Y chromosome (WD40 Y)
AAEL006156	N/A
AAEL013135	chromodomain helicase DNA binding protein
AAEL000922	polyribonucleotide nucleotidyltransferase
AAEL001311	N/A

**Table 3:** LC + MS/MS hits specific to DENV2-infected AaG2 cell supernatants 1 dpi and 7 dpi.

Identifier	Comment
AAEL007132	dynein heavy chain
AAEL000596	myosin
AAEL007041	low density lipoprotein receptor (ldl)
AAEL013174	N/A
AAEL002544	N/A
AAEL001773	N/A

We chose to investigate the six proteins found in DENV2-infected Aag2 cell supernatants at both 1 and 7 dpi. Three of the proteins (SeqIDs, AAEL013174, AAEL002544, and AAEL01773) lacked homology to any known protein and lacked predicted signal peptides according to SignalP4.1 and were eliminated from further analysis. The remaining three proteins were annotated as dynein heavy chain (AAEL007132), myosin (AAEL000596), and low-density lipoprotein receptor (ldl) (AAEL007041). Although each of these proteins also lacked signal peptides according to SignalP4.1, previous literature has characterized protein-protein interactions between dynein heavy chain and myosin with flavivirus structural proteins suggesting that these proteins may end up in the supernatant during shedding of progeny virions or through secretion of exosomes^27^. We therefore chose to further investigate the role of AAEL007041 in DENV infection.

### DENV infection reduces LRP-1 protein expression

Despite its annotation, BLAST analysis of AAEL007041 revealed that this protein is homologous to low-density lipoprotein receptor-related protein 1 (LRP-1) rather than low-density lipoprotein receptor (LDLR). LRP-1 belongs to a group of cell surface proteins that can undergo regulated intramembrane proteolysis (RIP) upon ligand binding in a Notch-like fashion. LRP-1 proteolysis leads to the release of both intracellular and extracellular domains^16^. Additionally, LRP-1 has been identified as a factor that reduces accumulation of intracellular cholesterol^17,18,21–25^. We hypothesized that DENV infection modifies LRP-1 protein expression, which would impact intracellular cholesterol levels. We first confirmed that the two LRP-1 peptides we detected by LC + MS/MS resided in the approximately 500 kDa extracellular domain. Both peptides (i.e, CVGIDNFLMYSIGHQLK and ADYDGRNRV) were located in the 500 kDa extracellular domain of LRP-1. We then determined if LRP-1 expression is altered at the transcriptional level. DENV infection did not alter LRP-1 gene expression 1 or 7 dpi (Fig. 2B,C). Our result is consistent with previously published RNASeq data that determined the impact of DENV infection on gene expression in *Ae. aegypti* carcass, midgut, and salivary gland tissue^12^. In contrast, Western blot analysis using a monoclonal antibody against an evolutionarily conserved epitope in the 85 kDa intracellular LRP-1 domain revealed that DENV2 infection reduced LRP-1 protein expression (Fig. 2D). These results indicate that DENV infection reduces LRP-1 protein expression while increasing peptides that correspond to LRP-1’s extracellular domain.

### Diverse stimuli reduce LRP-1 protein expression in mosquito cells

**Figure 3:**
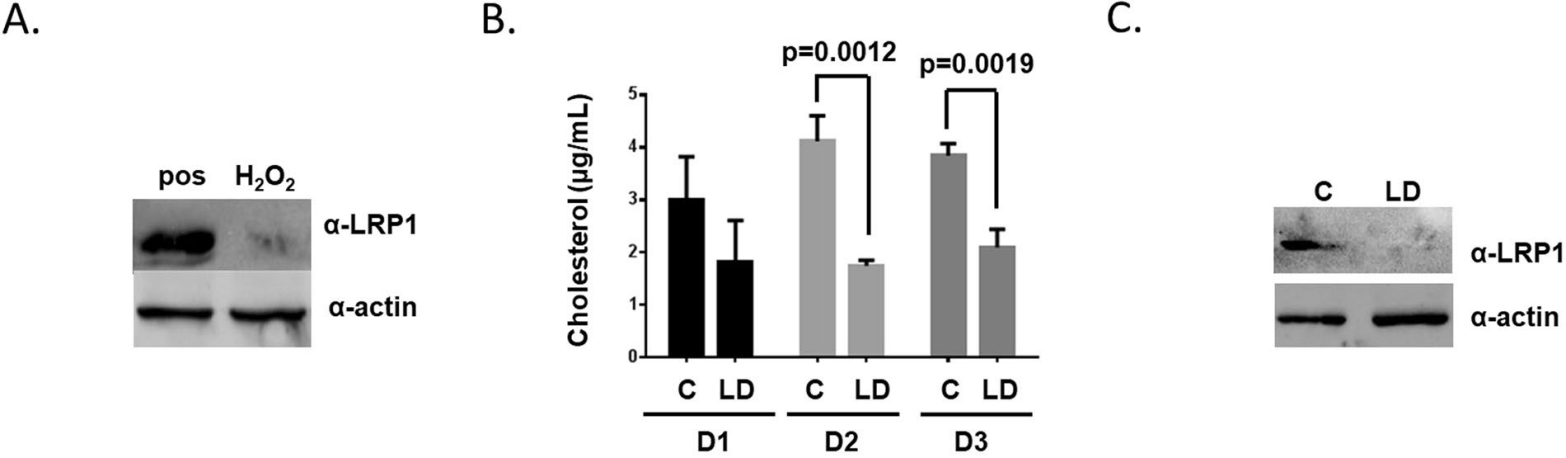
Diverse cellular stimuli reduce LRP-1 protein expression. (**A**) Western blot analysis of LRP-1 and actin in Aag2 cell lysates treated for six-hours with 1 mM hydrogen peroxide. (**B**) Cholesterol content was determined in Aag2 cells treated with complete (**C**) and lipid-depleted (LD) media 1, 2, and 3 dpt. The experiment was performed in triplicate and Student’s t tests were performed between groups to assess statistical significance. (**C**) Western blot analysis of LRP-1 and actin in Aag2 cells treated for three days with lipid-depleted media.

Previous research identified a number of physiologic stimuli that can reduce LRP-protein expression, and this process often occurs through RIP. Both reactive oxygen species and cholesterol depletion can reduce LRP-1 protein expression^19,28–32^. Flavivirus infection is known to lead to the production of reactive oxygen species and it modifies intracellular cholesterol^33–37^. We determined if reactive oxygen species and lipid depletion could reduce LRP-1 protein expression in Aag2 cells. Hydrogen peroxide was used as a prototypical reactive oxygen species that is induced during DENV infection. A six-hour treatment with 1 mM hydrogen peroxide reduced LRP-1 protein expression (Fig. 3A). We also tested if treatment of Aag2 cells with lipid-depleted media reduced intracellular cholesterol concentration after 1, 2, and 3 days. Intracellular cholesterol concentration was significantly reduced 2 and 3 days post treatment (Fig. 3B). A three-day treatment with lipid-depleted media reduced LRP-1 expression (Fig. 3C). These data support that cellular stimuli that occur during flavivirus infection can reduce LRP-1 protein expression.

**Figure 4:**
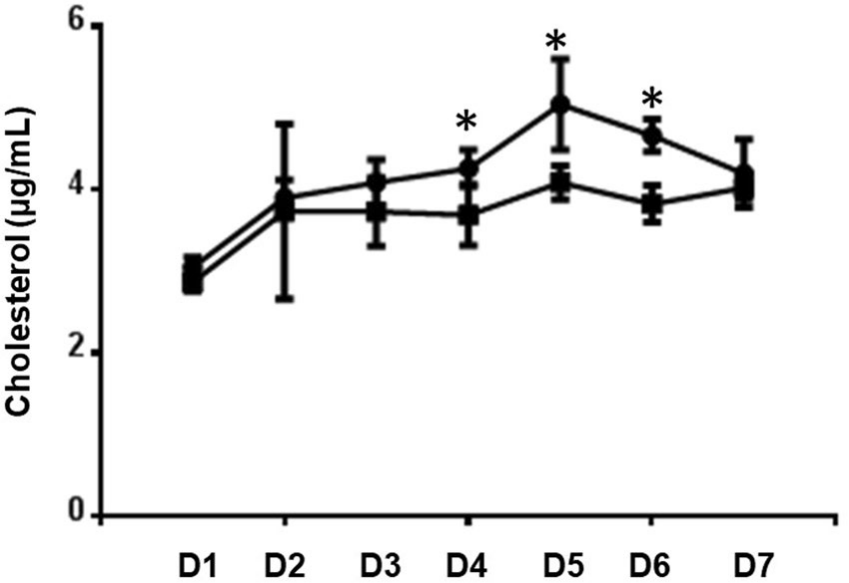
Cholesterol content in uninfected and infected cells. Cholesterol content was determined in mock (circles) and DENV-infected (squares) Aag2 cells each day for seven days. The experiment was performed in triplicate and Student’s t tests were performed between groups to assess statistical significance.

We then determined if DENV infection creates a cellular environment that would mimic lipid depletion by quantifying the cholesterol content in uninfected and infected cells each day during a 1-week time course. We found that DENV infection limited intracellular cholesterol accumulation similar to our treatment with lipid-depleted media (Figs 3C and 4). These data reveal that DENV infection reduces cholesterol accumulation in mosquito cells, which is a stimulus that can reduce LRP-1 protein expression.

### LRP-1 decreases intracellular cholesterol content and inhibits DENV replication

**Figure 5:**
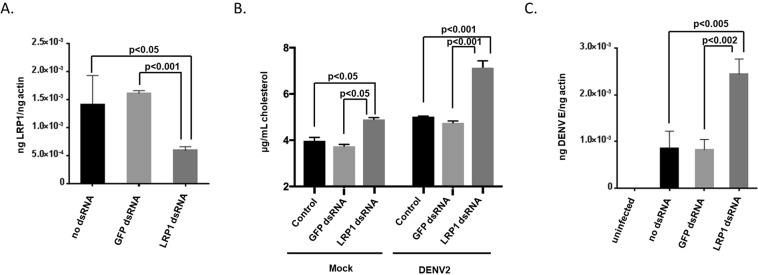
LRP-1 knockdown increases intracellular cholesterol and DENV viral RNA. (**A**) Total cellular RNA was extracted from Aag2 cells 24 hours after being either transfected with GFP dsRNA, or transfected with LRP1 dsRNA. A no dsRNA control was included. qRT-PCR was performed to determine LRP-1 gene expression and normalized to actin gene expression. (**B**,**C**) Aag2 cells were transfected with GFP or LRP-1 dsRNA. A no dsRNA control was included. Cells were then either Mock or DENV2-infected 24 hpt. (**B**) Cholesterol content was determined 24 hpi. (**C**) qRT-PCR was performed to determine intracellular DENV2 vRNA levels 24 hpi. Each experiment was performed in triplicate and Student’s t tests were performed between groups to assess statistical significance.

One function of LRP-1 is to limit the concentration of intracellular cholesterol^18,21,23–25^. We hypothesized DENV reduces LRP-1 protein expression to maintain intracellular cholesterol levels, which facilitates virus replication. In order to test this hypothesis, we transfected Aag2 cells with control or LRP-1 dsRNA and confirmed gene knockdown by qRT-PCR (Fig. 5A). We then transfected Aag2 cells with control or LRP-1 dsRNA, and then inoculated cells with DENV2. We assessed intracellular cholesterol content and total viral RNA 24 hours post infection (hpi). LRP-1 knockdown significantly increased intracellular cholesterol levels (Fig. 5B). Further, LRP-1 knockdown significantly increased total viral RNA levels (Fig. 5C). These data suggest that LRP-1 knockdown promotes intracellular cholesterol accumulation and that this correlates with increased virus replication.

**Figure 6:**
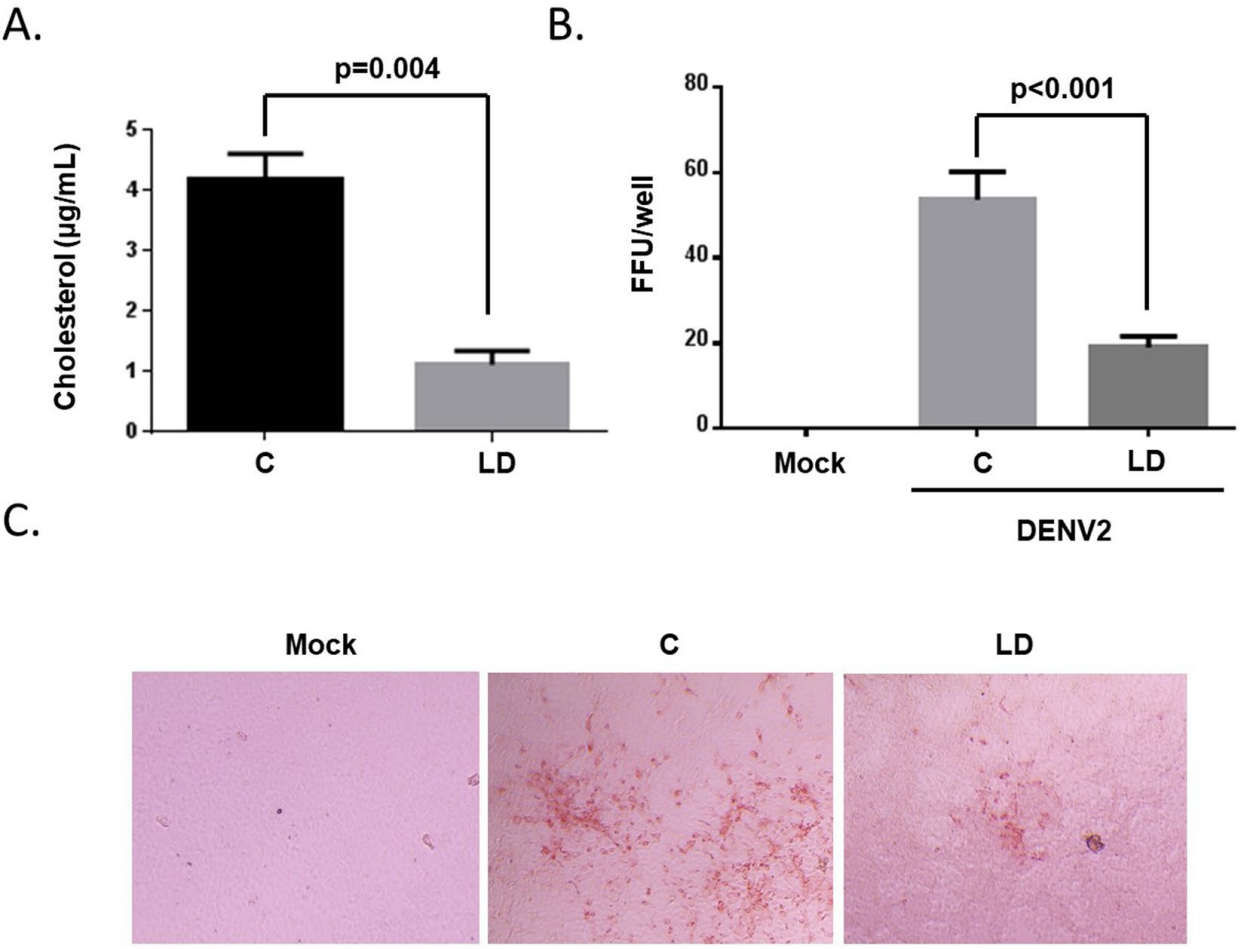
Lipid depletion reduces DENV infection. (**A**) Cholesterol content was determined in Aag2 cells treated with complete (**C**) and lipid-depleted (LD) media 7 dpt. (**B**) Aag2 cells treated with complete or lipid-depleted media for 7 days were inoculated with DENV2 in complete media for 1 hr. Unbound virus was removed and complete media was added for 3 days. Cells were fixed and DENV2-positive foci were revealed used DENV2-specific antibody. Foci were quantified in each well. Mock-infected controls were included. (**C**) Representative 20x images of the focus forming unit assay are shown. Experiments were performed in triplicate and Student’s t tests were performed between groups to assess statistical significance.

We confirmed that intracellular cholesterol is important for DENV infection by growing Aag2 cells in both complete and lipid-depleted media for 7 days. Cells grown in lipid-depleted media had significantly less intracellular cholesterol (Fig. 6A). Both control and lipid-depleted cells were inoculated with DENV in complete media, unbound virus was removed, and complete media was replaced onto the cells. A focus forming unit assay was performed 3 dpi and a statistically significant decrease in virus replication was observed on the lipid-depleted cells (Fig. 6B,C). These data confirm that intracellular cholesterol facilitates DENV infection.

### Aag2 cells grow in lipid-depleted media

**Figure 7:**
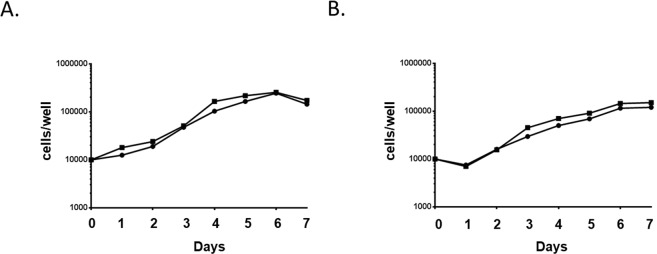
Aag2 cells grow with lipid-depleted media and during DENV2 infection. Cell growth of Aag2 cells was determined each day for 7 days in (**A**) complete and (**B**) lipid-depleted media. Cells were Mock (circles) or DENV2-infected (squares).

Our data suggest that DENV reduces LRP-1 protein expression in order to maintain intracellular cholesterol to facilitate its replication; however, we also found that Aag2 cells reduce LRP-1 protein expression when treated with lipid-depleted media (Fig. 3B,C). It is possible lipid depletion is also harmful to the cell, and that both the virus and the cell benefit by reducing LRP-1 protein expression. To test this hypothesis, we determined the growth kinetics of uninfected and infected Aag2 cells in the presence of complete and lipid-depleted media. We found that Aag2 cells grown in lipid-depleted media had a marginal growth disadvantage that was not statistically significant and that the growth kinetics did not change during infection (Fig. 7A,B). These data suggest that a reduction in host-derived intracellular lipids do not significantly impact cell growth.

## Discussion

DENV is one of the most important vector-borne human diseases, and has the potential to spread due to changes in climate and movement of humans and animals^38^. Few effective options are available to combat the spread of this disease. New or improved control strategies are needed^3^. There is increasing interest in the molecular mechanisms of DENV replication in its mosquito vector as a prophylactic or therapeutic target because of proof-of-principle studies showing that it is theoretically possible to target vector proteins or pathways that interfere with acquisition and transmission of vector-borne diseases^3–11,39–41^.

A number of recent studies have shown the importance of cholesterol homeostasis during DENV replication in *Ae. aegypti*^42–47^. For instance, *Wolbachia* infection perturbs intracellular cholesterol and vesicular trafficking and leads to inhibition of virus replication^47^. Further, altering cholesterol distribution by modifying expression of sterol carrier protein 2 inhibited DENV infection^42^. Our laboratory found that human low-density lipoprotein inhibits flavivirus infection *in vitro* and *in vivo*^46^. It is clear that DENV is dependent on cholesterol itself or lipid sorting pathways in the mosquito.

Cholesterol dependence is curious given that *Ae. aegypti* is a cholesterol auxotroph; although, this lipid is readily available in each blood meal^48^. Aag2 cells and the midgut epithelium are able to take up human LDL via clathrin-mediated endocytosis^46^. Cholesterol uptake appears to benefit oogenesis, although our current data suggests that providing cell with lipid-depleted media only marginally reduces cell growth rate^48^. *Ae. aegypti* cells are able to synthesize their own lipids from protein and sugar, so host lipids are likely not required for growth of somatic cells.

The current study identified extracellular fragments of *Ae. aegypti* LRP-1 that were only present during DENV2 infection. We confirmed that LRP-1 protein expression is reduced during infection, and during stimuli that occur during virus infection such as exposure to reactive oxygen species and changes in cholesterol concentration. Additional research is required to determine if the level of stimulus is equal during virus infection. DENV2 infection also limited cholesterol accumulation, which either occurred by promoting shedding of intracellular cholesterol or preventing cholesterol uptake.

LRP-1 has been identified as a factor that reduces accumulation of intracellular cholesterol^17,18,21–25^. We hypothesized that this function of LRP-1 would have an antiviral effect. We confirmed this hypothesis by performing RNAi and showed that LRP-1 knockdown increased both intracellular cholesterol content and DENV2 vRNA. We also showed that depletion of host lipids from Aag2 cells reduced DENV2 infection. A caveat to our study is that we have not directly linked the extracellular peptides that were identified by LC + MS/MS to RIP, which would require more research into structural alteration of Aedes aegypti LRP-1 during physiological stimuli and identification of enzymes responsible for its proteolytic cleavage. Unfortunately, the enzymes identified in vertebrate systems are not evolutionarily conserved in mosquitoes. Further research is required to understand the details of RIP in mosquito cells.

These data provide support that LRP-1 functions to reduce host-derived intracellular cholesterol content, and that DENV2 replication is enhanced when cholesterol levels are increased. Our data suggest that multiple stimuli that are present during virus infection can reduce LRP-1 protein expression and that this may be due to RIP. Future studies will reveal the molecular pathways that *Ae. aegypti* use to acquire and maintain host lipids and how these pathways benefit flaviviruses during acquisition and transmission.
